# Rhinoschleroma

**DOI:** 10.4269/ajtmh.14-0288

**Published:** 2015-01-07

**Authors:** Juan Carlos Cataño, Sabrina Gallego

**Affiliations:** Infectious Diseases Section, Internal Medicine Department, and Microsurgery and Oncology Plastic Surgery Department, University of Antioquia Medical School, Medellín, Colombia

## Abstract

A 39 year-old man came to our institution because of a five-year history of a progressive painful growing mass on his left nostril, which cause airway obstruction with ulceration. Because of a suspicion of malignancy, surgery (mass resection and subtotal nasal reconstruction) was performed. Histologic samples ruled out malignancy, and tissue cultures for fungus and mycobacteria were negative, but regular aerobic cultures were positive for *Klebsiella rhinoscleromatis*. The patient was given a six-month regimen of ciprofloxacin, and a dramatic improvement was observed.

A 39 year-old man who had lived his entire life in rural Colombia and who had no significant medical history except for a minor trauma in his left cheek and temporal region more than 10 years ago. He came to our institution with a history of five years of a growing painful mass on his left nostril, which caused airway obstruction. At physical examination, he had an erythematous mass with central ulceration, purulent discharge, adherent to deep tissues, producing partial obstruction of the left eye visual field, and complete obliteration of the left nostril (Figure 1A and B). Computed tomography of the face was performed and showed a mass that originated from the left middle turbinate, invaded the medium maxillary sinus and orbital wall, and extended to the intraocular fat in close contact with the ocular globe.

**Figure 1.**
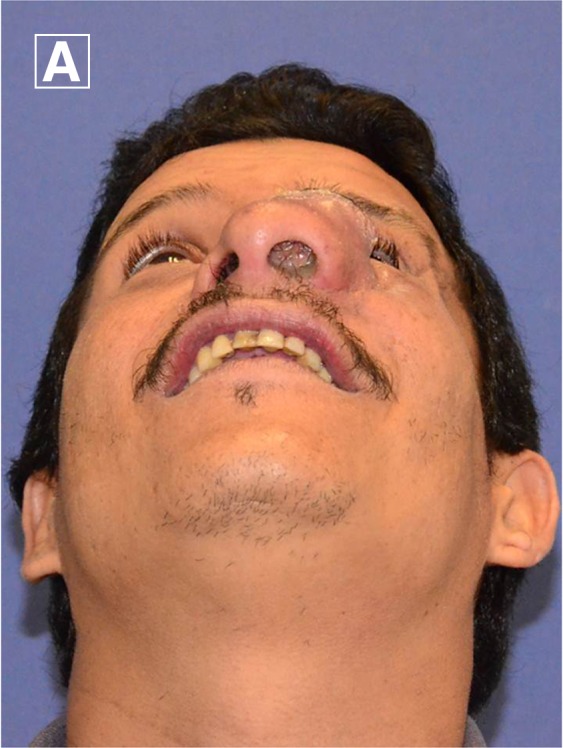

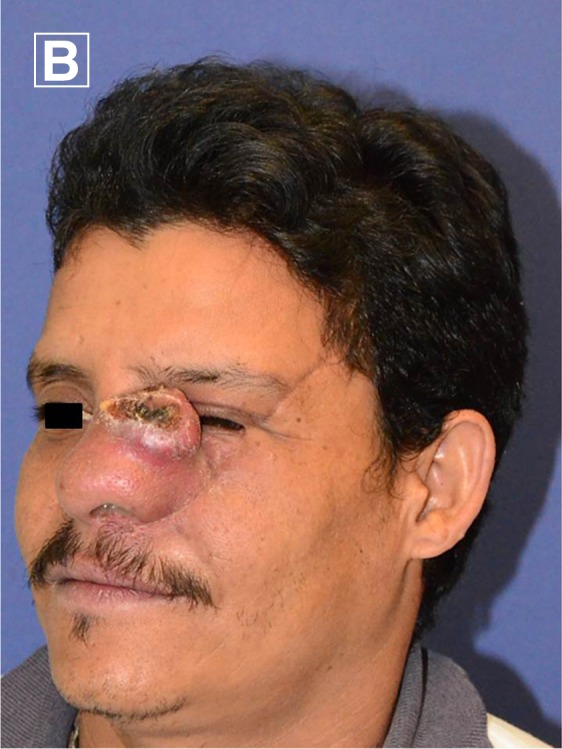

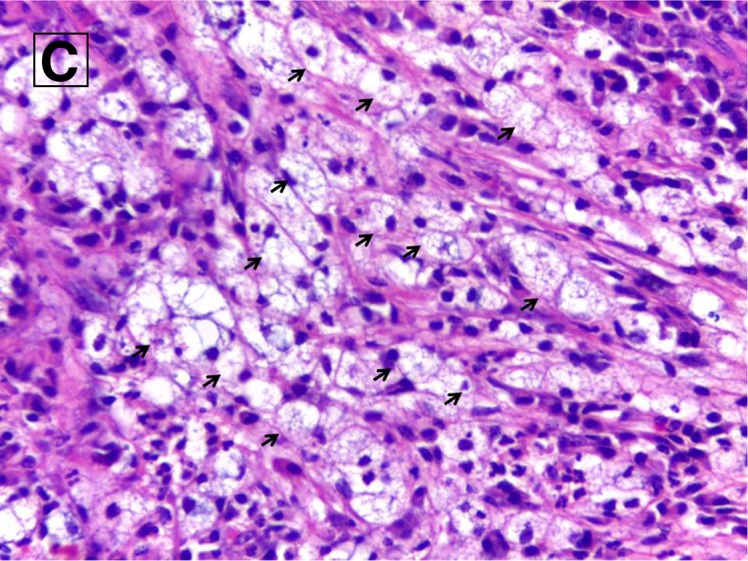

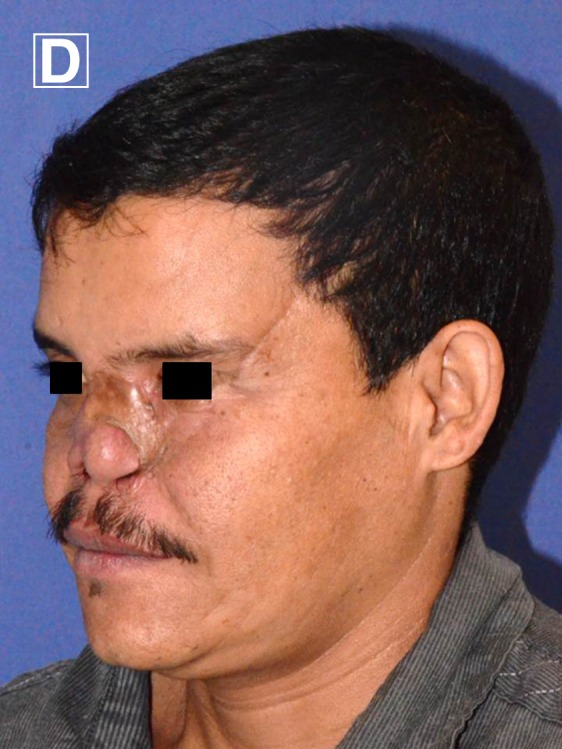
**A** and **B**, Patient with an erythematous mass with central ulceration, purulent discharge, adherent to deep tissues, producing partial obstruction of the left eye visual field, and complete obliteration of the left nostril. **C**, Histologic samples from the patient showing groups of large vacuolated histiocytes containing gram-negative bacteria (Mikulicz cells), many of which had Russell bodies (eosinophilic, homogenous inclusions found in a plasma cell undergoing excessive synthesis of immunoglobulin; haematoxylin and eosin 400×) (**arrows**). **D**, Patient after treatment at the recent follow-up before he was scheduled for a definitive nasal reconstruction.

A biopsy and culture were not performed. However, because of a suspicion of malignancy, a surgical mass resection was performed that included the left nasal bone, and the orbital and maxillary sinus wall; all orbital structures were preserve. The same day, a subtotal nasal reconstruction with an antebrachial free flap was performed. The patient showed an uneventful postoperative course.

Histologic samples from the mass and borders ruled out malignancy, but showed groups of large vacuolated histiocytes containing gram-negative bacteria (Mikulicz cells), many of which had Russell bodies (eosinophilic, homogenous inclusions found in a plasma cell undergoing excessive synthesis of immunoglobulin arrows (Figure 1C). Tissue cultures for fungus and mycobacteria were negative, but regular aerobic cultures were positive for *Klebsiella rhinoscleromatis*.

A six-month regimen of ciprofloxacin (750 mg every 12 hours) was started, and the patient showed dramatic improvement. At the most recent follow-up, there were no signs of relapse of infection, and the patient was scheduled for a definitive nasal reconstruction (Figure 1D).

